# Effect of Abandonment on Diversity and Abundance of Free-Living Nitrogen-Fixing Bacteria and Total Bacteria in the Cropland Soils of Hulun Buir, Inner Mongolia

**DOI:** 10.1371/journal.pone.0106714

**Published:** 2014-09-30

**Authors:** Huhe, Shinchilelt Borjigin, Yunxiang Cheng, Nobukiko Nomura, Toshiaki Nakajima, Toru Nakamura, Hiroo Uchiyama

**Affiliations:** 1 Graduate School of Life and Environmental Sciences, University of Tsukuba, Tsukuba, Ibaraki, Japan; 2 State Key Laboratory of Grassland Agro-Ecosystems, College of Pastoral Agriculture Science and Technology, Lanzhou University, Lanzhou, China; Radboud University Medical Centre, NCMLS, Netherlands

## Abstract

In Inner Mongolia, steppe grasslands face desertification or degradation because of human over activity. One of the reasons for this condition is that croplands have been abandoned after inappropriate agricultural management. The soils in these croplands present heterogeneous environments in which conditions affecting microbial growth and diversity fluctuate widely in space and time. In this study, we assessed the molecular ecology of total and free-living nitrogen-fixing bacterial communities in soils from steppe grasslands and croplands that were abandoned for different periods (1, 5, and 25 years) and compared the degree of recovery. The abandoned croplands included in the study were natural restoration areas without human activity. Denaturing gradient gel electrophoresis and quantitative PCR (qPCR) were used to analyze the *nifH* and 16S rRNA genes to study free-living diazotrophs and the total bacterial community, respectively. The diversities of free-living nitrogen fixers and total bacteria were significantly different between each site (*P*<0.001). Neither the total bacteria nor *nifH* gene community structure of a cropland abandoned for 25 years was significantly different from those of steppe grasslands. In contrast, results of qPCR analysis of free-living nitrogen fixers and total bacteria showed significantly high abundance levels in steppe grassland (*P*<0.01 and *P*<0.03, respectively). In this study, the microbial communities and their gene abundances were assessed in croplands that had been abandoned for different periods. An understanding of how environmental factors and changes in microbial communities affect abandoned croplands could aid in appropriate soil management to optimize the structures of soil microorganisms.

## Introduction

Steppe grasslands are distributed over vast areas in the arid and semiarid regions of the Eurasian continent [1]. The Inner Mongolia steppe is an important part of Eurasia and has been used by pastoral nomads for long periods of time. However, vast areas were converted to cropland, and farmers have increased the size of these croplands during the past 40 years because of a rapid increase in the human population. Subsequently, many of the croplands were abandoned because of soil degradation and desertification caused by inappropriate agricultural management [2], [3]. Further, some croplands were abandoned to restore natural vegetation, as in the case where the Chinese government proposed to restore farmlands to grasslands or forests [4]. Some plant species, including grasses, shrubs, and trees, are introduced into the abandoned croplands to restore the vegetation in some locations [5]–[8]. In most areas, the plant community of abandoned cropland is likely to be restored naturally by controlling human activities [9]. Many researchers have evaluated the abandoned cropland ecosystem changes by studying the vegetation changes [9]–[12]. There are interactions between plants and microorganisms. Plants exude diverse compounds, such as organic acids, enzymes, and polysaccharides, from the roots. Further, plants can recognize microbe-derived compounds and adjust their defense and growth responses according to the type of microorganisms encountered. Conversely, microorganisms can detect suitable plant hosts and initiate their colonization strategies in the rhizosphere by producing canonical plant growth-regulating substances such as auxins or cytokinins [13]. However, most studies have not evaluated changes in soil microbial community and its gene abundance in croplands that have been abandoned for different periods of time.

Cultivated soil or grassland soil contains an estimated 2×10^9^ prokaryotic cells per gram [14]. Soil microbial communities are important factors of agriculturally managed systems because they are responsible for most nutrient transformations in soil and influence the aboveground plant diversity and productivity [15]. Next to water, nitrogen is the second-most limiting factor for plant growth [16]. Nitrogen cycling in natural ecosystems and during traditional agricultural production rely on nitrogen fixation of diazotrophic bacteria [17]. Diazotrophs are highly diverse and widely distributed across bacterial and archaeal taxa [18]. Approximately 80% of biological nitrogen fixation is performed by diazotrophs in symbiosis with legumes [19]. However, under specific conditions, the free-living bacteria in soil (e.g., cyanobacteria, *Pseudomonas*, *Azospirillum*, and *Azotobacter*), may fix significant amounts of nitrogen (0–60 kg·N·ha^−1^·year^−1^) [20], [21]. This may be particularly important in abandoned field soils, where legume plants have not been cultivated and there are very few symbiotic plants (e.g., Leguminosae, *Azolla*, *Myrica*, and *Alnus*.).

In this study, the diazotrophic population was monitored by PCR-denaturing gradient gel electrophoresis (DGGE) exploiting the *nifH* gene. The *nifH* gene is the most conserved gene in the *nif* operon and encodes the Fe subunit of the nitrogenase enzyme [22]. Because of the conserved nature of the *nifH* gene, it has been possible to identify primer sets that can be used to analyze nitrogen fixers so that this community can be analyzed by a PCR-DGGE–based technique [20], [23]–[25]. We have tested the diversity and abundances of free-living nitrogen fixers and the total bacterial population changes that occurred over time in soils belonging to artificially disrupted environments (abandoned cropland soils).

## Materials and Methods

### Ethics statement

No specific permissions are required for our conducting field survey in this area, since land in China belongs to the public and our field studies did not involve any endangered or protected plant species within.

### Site description and sample collection

**Figure 1:**
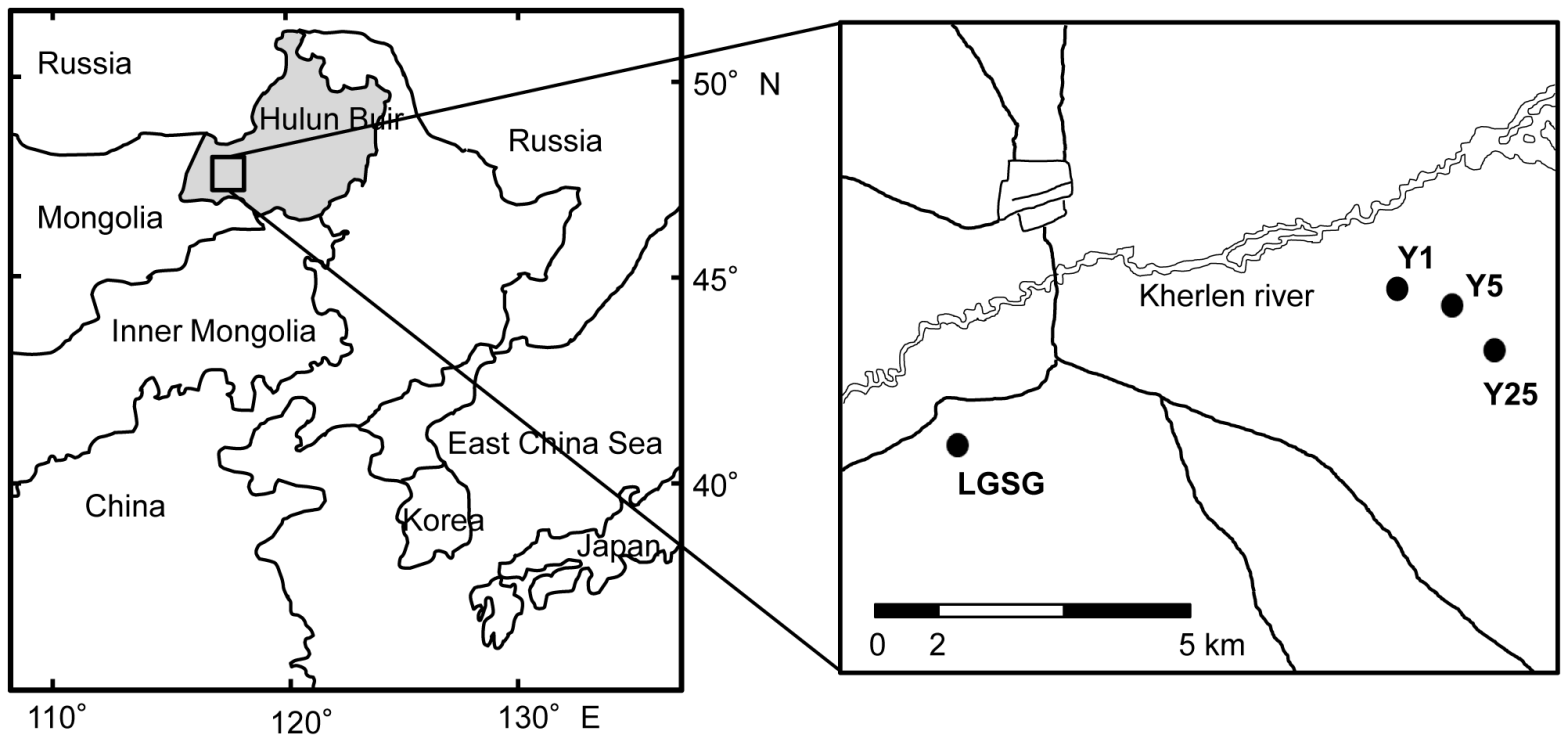
Map of the study area and the 4 study sites (•) in Hulun Buir.

The study area is located in the Hulun Buir grassland (115°31′–126°04′ E, 47°05′–53°20′ N) in northeastern Inner Mongolia, China (Figure 1). The Hulun Buir grassland area is about 2.6×10^5^ km^2^, with a west to east distribution of arid steppe, semi-arid steppe, and meadow steppe. The study area is located in the semi-arid areas.

We established 4 sites, 3 abandoned croplands and a light-grazing steppe grassland (LGSG) (Figure 1) that had an intensity of about 1.4 sheep ha^−1^. The 3 croplands were abandoned for 1, 5, and 25 years (Y1, Y5, and Y25, respectively), and the control area was a LGSG. In the Y1, Y5, and Y25 sites, *Zea mays*, *Helianthus annuus*, and *Elymus cylindricus* were rotated for approximately 40 years. The sites were subsequently abandoned because of land degradation; soil fertility including both organic C and total N had decreased by approximately 70%.

Plant surveying and soil sampling were conducted in August 2010. All of the sites were selected for their similar topography (flat). Each site contained 5 replicates in a randomized plot (1×1 m) design: Y1 (site 1), plots 1–5; Y5 (site 2), plots 6–10; Y25 (site 3), plots 11–15; and LGSG (site 4), plots 16–20. The coordinates and elevations of the sampled sites are as follows: Y1, 48° 38′ 43″ N, 116° 57′ 56″ E, 545 m; Y5, 48° 38′ 50″ N, 117° 00′ 48″ E, 550 m; Y25, 48° 38′ 45″ N, 117° 01′ 56″ E, 545 m and LGSG, 48° 32′ 00″ N, 116° 40′ 18″ E, 568 m. The mean temperature and precipitation from 2000 to 2009 for each site [26] were as follows: LGSG, Y1, Y5, and Y25, 1.6°C and 213 mm. In each plot, the species composition was recorded. Plant communities were classified on the basis of their differential species [27], [28]; all species were identified and measured for cover, height, and density, and Shannon-Wiener diversity index was calculated. Soil moisture was measured with a TRIME-FM (Ettlingen, Germany). Above-ground plant biomass was also determined by clipping the plants at ground level, sorting by species, drying at 60°C for 48 h, and weighing the samples (Table 1 and Table S1).

**Table 1:** Changes to pH, available NO_3_-N, available NH_4_-N, available P, soluble Fe, organic C, total N, plant diversity (P-*H′*), plant biomass (P-B), soil moisture, and hydraulic conductivity (HC) across the field trial and Pearson's product-moment correlation analysis comparing data to *nifH* and 16S rRNA diversity and gene copies.

Abandoned Cropland or significance parameter	pH	NO_3_-N (mg kg ^−1^)	NH_4_-N (mg kg ^−1^)	P (mg kg ^−1^)	Fe (g kg ^−1^)	Organic C (g kg ^−1^)	Total N (g kg ^−1^)	H_2_O (%)	P-*H*′	P-B	HC (×10^−3^ cm s^−1^)
Abandoned Cropland*											
Y1	7.72±0.13 a	3.35±0.42 a	1.59±0.64 a	12.38±4.09 a	0.43±0.06 a	6.55±0.96 a	0.7±0.09 a	8.0±0 a	1.63±0.34 a	48.5±9.77 a	2.93±0.07 a
Y5	8.57±0.37 b	3.99±0.67 b	0.76±0.04 a	12.92±2.68 a	0.56±0.04 a	9.16±1.51 a	0.94±0.12 b	9.06±0.13 ab	1.71±0.43 a	17.16±4.44 b	2.95±0.03 a
Y25	7.82±0.17 a	3.54±0.53 a	1.09±0.21 a	12.5±1.8 a	0.46±0.04 b	7.23±0.64 a	0.75±0.06 ab	10.7±1.09 b	1.82±0.21 a	79.65±13.5 c	2.79±0.02 b
LGSG	6.21±0.14 c	4.21±0.06 c	4.84±0.37 b	24.48±5.67 b	1.12±0.06 c	24.84±3.09 b	2.3±1.71 c	12.12±1.4 bc	0.94±0.02 b	141.9±5.54 d	2.75±0.02 b
Correlation (*P* **)											
with *nifH* DGGE *H*′	+++	++	NS	+++	NS	NS	NS	NS	NS	NS	NS
with *nifH* copy number	NS	NS	++	+++	+++	+++	++	+++	—	++	NS
with 16S rRNA DGGE *H*′	NS	NS	NS	NS	+++	++	++	+++	NS	++	---
with 16S rRNA copy number	NS	NS	+++	NS	NS	++	++	NS	NS	++	NS

*Y1, Y5 and Y25 mean field abandoned for 1 year, 5 years and 25 years, respectively. LGSG is light grazing steppe grassland. The values shown for management factors are means ± standard errors.

**P, Pearson's product-moment correlation coefficient; NS, not significant; ++/—, significant positive or negative correlation at *P*<0.05; +++/---, significant positive or negative correlation at *P*<0.01. Significant differences are indicated by different letters.

In each plot, the soil samples were collected from 5 randomly selected points (0 to 10 cm deep) and mixed into 1 sample. After carefully removing the surface organic materials and fine roots, each mixed sample was divided into 2 parts. One part was air-dried for the analysis of soil physicochemical properties. The other was sifted through a 2-mm sieve, sealed in sample vials, kept on ice for transport to the laboratory, and stored at −20°C for microbial assays.

The soil texture was determined by mechanical analysis using the pipette method [29], and the soil texture was used to estimate the saturated hydraulic conductivity [30] for each site (Table S2). The soil texture was classified according to the International Society of Soil Science (ISSS) classification system. The soils of all 4 sites were sandy loam.

Concentrations of NO_3_-N and NH_4_-N in the KCl extracts were determined with the zinc reduction-naphthylethylenediamine method for NO_3_-N [31] and the indophenol blue colorimetric method for NH_4_-N [32]. The soil phosphorus content was determined by the Truog method [33]. Ferrous iron was measured by the *o*-phenanthroline method [34], [35]. The organic C and total N were determined by the dry combustion method using an NC analyzer (Sumigraph NC-900; Sumika Chemical Analysis Service, Tokyo, Japan). The soil samples were previously treated with acid to eliminate water and inorganic carbonates [36]. The soil pH was obtained by measuring the equilibrium pH of soil pastes containing 1 g of soil homogenized in 1 mL of H_2_O (Table 1).

### DNA extraction and PCR

DNA from each of the 20 samples (4 sites×5 replicates) was extracted in 3 subsamples from 0.5 g of soil with the FastDNA Spin Kit for soil (MP Biomedicals, Illkirch, France) according to the manufacturer's protocol. The quality and quantity of the DNA extracts were checked with a SmartSpec Plus spectrophotometer (Bio-Rad Laboratories, United States). The samples were pooled and stored at -20°C until use.

A fragment of the *nifH* gene (approximately 360 bp) was amplified with a nested PCR strategy. First-round reactions were performed with the primers nifH32F (TGAGACAGATAGCTATYTAYGGHAA) and nifH623R (GATGTTCGCGCGGCACGAADTRNATSA) as described previously [37]. The genomic DNA extract (15–40 ng) was added to PCR mixtures containing 5 µL of 10×ExTaq buffer (Takara, Madison, WI, United States), 4 µL of a mix of deoxynucleoside triphosphates (2.5 mM each), 37.5 µL of water, 0.5 µL of 100 µM nifH32F, 0.5 µL of 100 µM nifH623R, and 0.5 µL of ExTaq DNA polymerase (5 U/µL; Takara, Madison, WI, United States). The reaction mixtures were amplified by 1 denaturation step (5 min at 94°C), followed by 30 cycles of 94°C for 1 min, 50°C for 1 min, and 72°C for 1 min, and 1 final 7 min extension cycle at 72°C. For the second round of the nested amplification, 1 µL of this reaction mixture was used as the template in a 50 µL reaction mixture containing the same reagent mixture described above, but with 39 µL of water, 0.25 µL of 100 µM nifH1-GC (in order to clamp the products for DGGE, the primer nifH1 [CTGYGAYCCNAARGCNGA] was added to the GC-clamp [CGCCCGCCGCGCGCGGCGGGCGGGGCGGGGGCACGGGGGG] on the 5′ side), and 0.25 µL of 100 µM nifH2 (ADNGCCATCATYTCNCC) [38]. The thermal cycling protocol for the nested reactions was the same as above except that the annealing temperature was raised to 57°C.

Fragments of approximately 200 bp, corresponding to the V3 region of the 16S rRNA gene [39], were amplified using a reaction mixture that contained the same reagent mixture described above, except with 0.25 µL of 100 µM 357F-GC (CCTACGGGAGGCAGCAG-GC-clamp) and 0.25 µL of 100 µM 518R (GTATTACCGCGGCTGG); products were amplified using a touchdown thermocycling program [39]. All of the PCR amplicons were electrophoresed on an agarose gel to ascertain the sizes and purified using the UltraClean PCR Clean-Up Kit (MO BIO Laboratories, Carlsbad, CA, United States).

### DGGE

DGGE was performed using the D-Code system (Bio-Rad Laboratories, Hercules, CA, United States) as described by Baxter and Cummings [40]. The polyacrylamide concentration in the gel was 8%, and the linear denaturing gradient was 30% to 60% (100% denaturant corresponds to 7 M urea and 40% deionized formamide). The gel was run at 36 V for 18 h at 60°C in 0.5×TAE buffer. The gel was then stained for 30 min with 1∶10,000 (v/v) SYBR Gold, rinsed with 0.5×TAE, and scanned on a transilluminator. Bands were identified, and relative intensities were calculated based on the percentage of intensity of each band in a lane. This was done with an image-analyzing system (Image Master; Amersham Pharmacia Biotech, Uppsala, Sweden). Shannon-Wiener diversity index (*H*′) was calculated by the formula *H*′ = −Σ *p_i_* ln(*p_i_*), where *p_i_* is the ratio of relative intensity of band *i* compared with the relative intensity of the lane. The used of different gradient gel (30% to 70%) to assessed the reproducibility of the DGGE results, similar results were obtained (data not shown).

### Real-time PCR assay

Reactions were set up using SYBR green (Bio-Rad Laboratories, the Netherlands) according to Baxter and Cummings [41] with the LightCycler 1.5 system (Roche Applied Sciences, Indianapolis, IN, United States). Reaction mixtures were heated to 95°C for 15 min to denature the DNA before completing 40 cycles of denaturation (95°C for 45 s/15 s [*nifH*/16S rRNA]), annealing (55°C for 45 s/65°C for 15 s [*nifH*/16S rRNA]), and extension (72°C for 45 s/15 s [*nifH*/16S rRNA]). Soil DNA extracts were diluted 1∶100 to prevent inhibition of PCR by soil contaminants (e.g., by co-extracted humic substances), and each run included triplicate reactions for each DNA sample, the standard curve, and the no template control. The average copy number was converted into copies of the gene per gram of soil. nifHF (AAAGGYGGWATCGGYAARTCCACCAC) and nifHR (TTGTTSGCSGCR TACATSGCCATCAT) primers [42] were used for *nifH* quantitative PCR (qPCR), and 357F and 518R primers were used for total bacteria qPCR. Dilution series of pGEM-T Easy vector (Promega, Madison, WI, United States) DNA with cloned bacterial *nifH* (*Azospirillum brasilense* ATCC 29729) and 16S rRNA gene (*Pseudomonas aeruginosa* PAO1) fragments were used to generate standard curves ranging from 10^1^ to 10^7^ gene copies·µL^−1^ for DNA quantification. The specificity of the amplified products was checked by the observation of a single melting peak and the presence of a unique band of the expected size in a 2% agarose gel stained with ethidium bromide. The standard curve produced was linear (*r* ^2^>0.98), and the PCR efficiency (Eff = 10^(−1/slope)^-1) was>0.90.

### Statistical analysis

In all tests, significant effects/interactions were those with a *P* value that was <0.05. Statistical analysis was performed using SPSS statistical software package (version 19.0; SPSS, Inc., Chicago, IL, United States). Variables of each group must be normally distributed to perform Pearson's product-moment correlations and analysis of variance (ANOVA). The normal distribution of the residuals was evaluated using the Kolmogorov-Smirnov test. If the requirement was not met, data were log-transformed prior to analysis. The homogeneity of the variances was checked by Levene's test. For pairwise comparison of means, Tukey's test was applied. Differences between main effects were tested by ANOVA. Correlations between *nifH* and 16S rRNA diversity and gene copies and environmental factors were tested by Pearson's product-moment correlations.

The choice between a linear or unimodal species response model depends on the underlying gradient length, which is measured in standard deviation units along the first ordination axis and can be estimated by detrended correspondence analysis (DCA). It is recommended to use linear methods when the gradient length is <3, unimodal methods when it is>4, and any method for intermediate gradient lengths [43]. The DCA gradient length for *nifH* gene patterns was 1.77, and that for 16S rRNA patterns was 1.11. Therefore, linear species response models such as partial least-squares regression (PLSR) and redundancy analysis (RDA) were used for multivariate statistical analysis. PLSR is an extension of multiple regression analysis in which the effects of linear combinations of several predictors on a response variable (or multiple response variables) are analyzed. PLSR is especially useful when the number of predictor variables is similar to or higher than the number of observations and/or predictors are highly correlated [44], [45]. For PLSR model generation, the software assigns a value known as the variable influence on projection (VIP) to each environmental variable. The VIP indicates the relative importance to the model. Significance was assessed using the VIP parameter (a VIP>1 indicated a significant contribution of the environmental factor to the statistical model) [46], [47]. RDA can be considered an extension of principal component analysis (PCA) in which the main components are constrained to be linear combinations of the environmental variables. RDA not only represents the main patterns of species variation as much as they can be explained by the measured environmental variables but also displays correlations between each species and each environmental variable in the data [48]. DCA, RDA, and PCA were performed using the Canoco program for Windows 4.5 (Biometris, Wageningen, the Netherlands). PLSR was performed using the Simca-P 11.0 (Umetrics AB, Umea, Sweden).

## Results

### Diversity of *nifH*

DGGE gels are shown in Figure S1A. Analysis of the *nifH* DGGE Shannon-Wiener diversity index values for the whole data set indicated that the abandoned period significantly affected the *nifH* diversity (*P*<0.001) by a separate ANOVA. For these reasons, multiple comparisons of the *nifH* DGGE Shannon-Wiener diversity index values were conducted.

**Figure 2:**
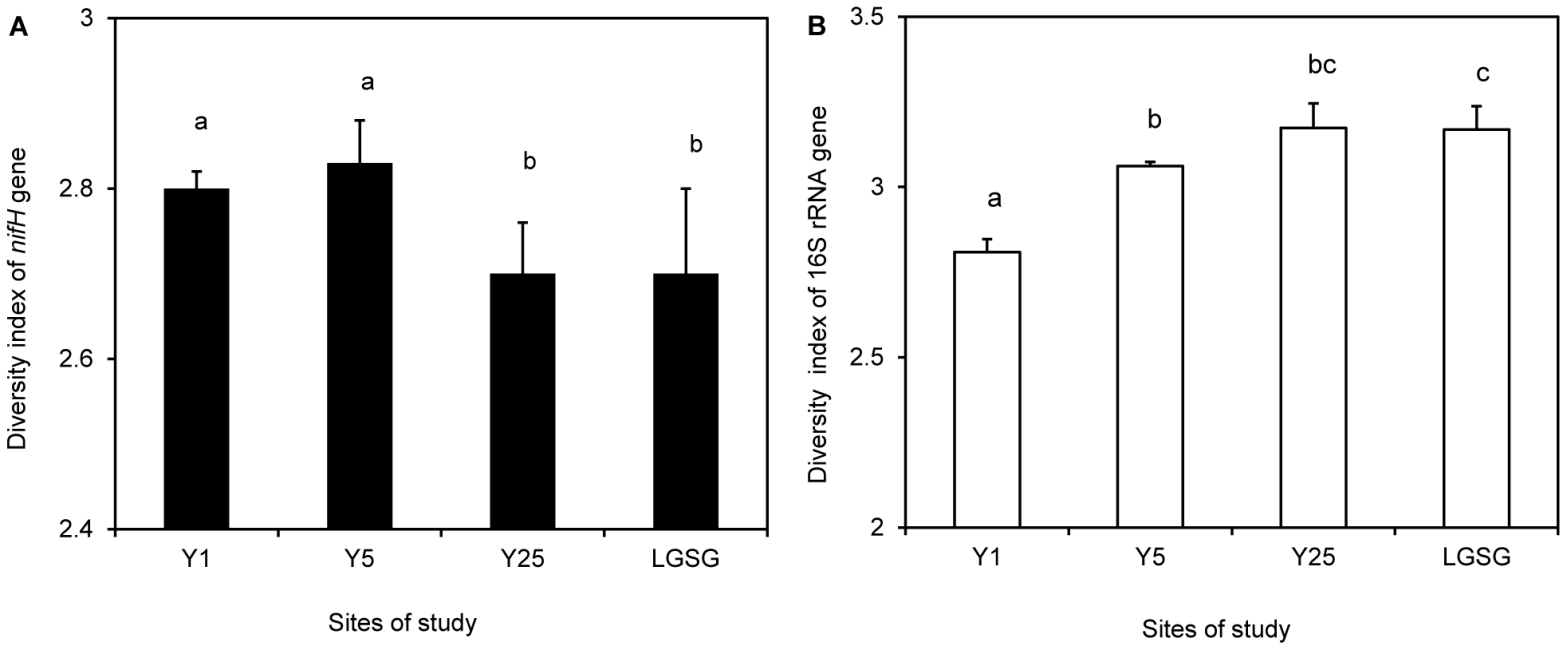
Shannon-Wiener diversity index values for *nifH* (A) and 16S rRNA genes (B). Data sets and results of analysis of variance (ANOVA) in the abandoned cropland (Y1, Y5, and Y25) and light-grazing steppe grassland (LGSG) soils (n = 5; error bars represent standard deviations). Significant differences are indicated by different letters.

Soils of Y1 (2.8±0.02) and Y5 (2.83±0.06) showed significantly higher *nifH* diversity than soils of Y25 (2.72±0.08) and LGSG (2.72±0.1) (Y1×Y25, *P* = 0.04; Y1×LGSG, *P* = 0.02; Y5×Y25, *P* = 0.009; Y5×LGSG, *P* = 0.025) (Figure 2A). The Y25 and LGSG soils did not show significant differences in *nifH* diversity. Pearson's product-moment correlation indicated that pH, NO_3_-N, and P were positively correlated with the *nifH* Shannon-Wiener diversity index (Table 1).

### qPCR of the *nifH* gene

**Figure 3:**
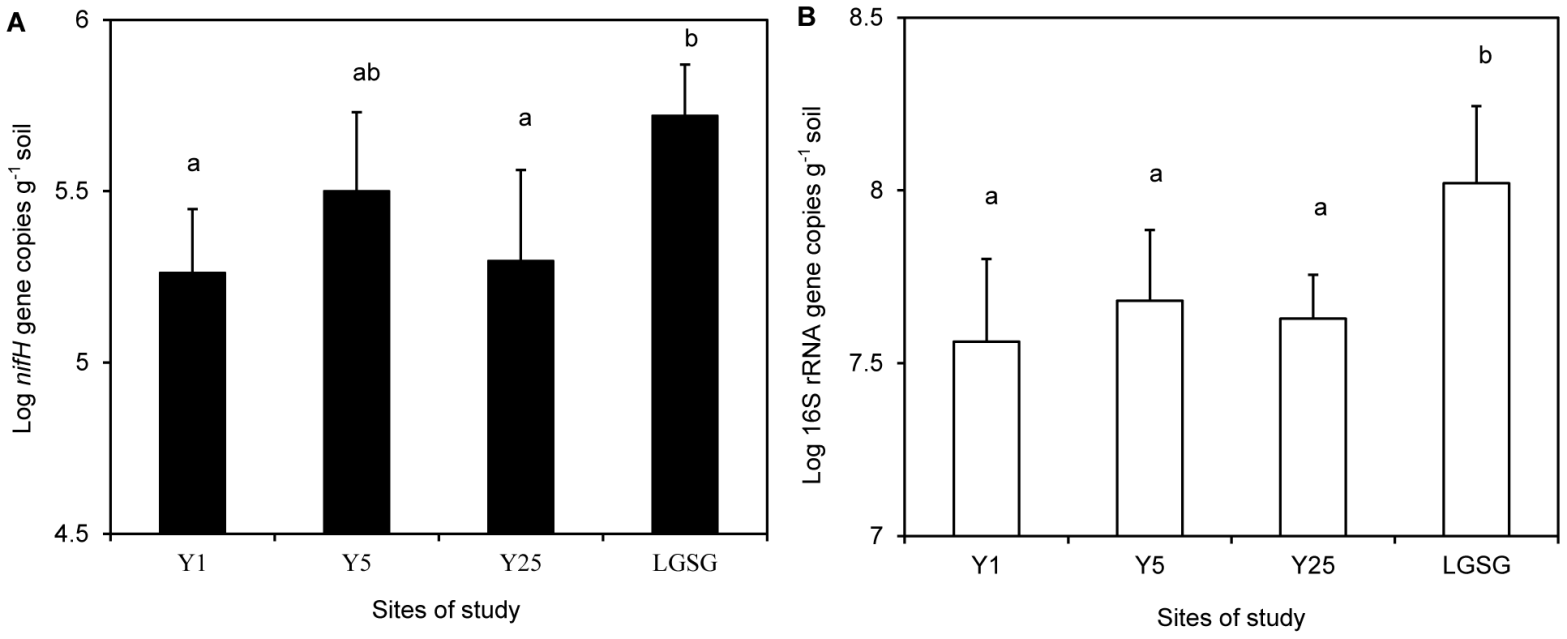
Copy numbers of the *nifH* (A) and 16S rRNA (B) genes. Results of analysis of variance (ANOVA) in the abandoned cropland (Y1, Y5, and Y25) and light-grazing steppe grassland (LGSG) soils (n = 5; error bars represent standard deviations). Significant differences are indicated by different letters.

We used qPCR to compare copy numbers of the functional gene (*nifH*) at the 4 sites (Figure 3A). The *nifH* gene copy number in LGSG soil (5.5×10^5^ copies·g^−1^ of soil) was the higher compared to the soils of the other 3 abandoned croplands (average number of copies·g^−1^ of soil, 1.9×10^5^ in Y1, 3.4×10^5^ in Y5, and 2.3×10^5^ in Y25). In the abandoned cropland soils, the *nifH* gene copy number did not significantly change over time (*P*>0.05) (Figure 3A).Using Pearson's product-moment correlation, we found that NH_4_-N, P, Fe, organic C, total N, soil moisture, and plant biomass were positively correlated, and the Shannon-Wiener diversity index was negatively correlated with the *nifH* copy number (Table 1).

### Total bacterial diversity

There are clearly differences in the nitrogen-fixing communities of soil from long and short abandoned periods. In order to ensure that these factors are affecting the nitrogen-fixing community specifically and not the bacterial community as a whole, the 16S rRNA gene diversity and abundance were also analyzed. DGGE gels showing the diversity of total bacteria are shown in Figure S1B. As with the free-living nitrogen-fixing community, ANOVA results for the Shannon-Wiener diversity index of the 16S rRNA gene indicated that the abandoned period (*P*<0.001) was a significant factor. Therefore, each sample was analyzed separately by multiple comparisons. Soils of Y25 and LGSG (both almost equal, 3.17±0.07) showed higher 16S rRNA diversity than soils of Y1 (2.8±0.04) and Y5 (3.06±0.01) (Figure 2B). Pearson's product-moment correlation indicated that organic C, total N, Fe, soil moisture, and plant biomass were positively correlated, and hydraulic conductivity was negatively correlated with the 16S rRNA gene Shannon-Wiener diversity index (Table 1).

### qPCR of the 16S rRNA gene

By performing the qPCR analysis, we aimed to compare the differences between each site rather than attain an absolute quantification. Similar to *nifH* results presented above, the gene copy numbers of 16S rRNA in LGSG soil (1.2×10^8^ copies·g^−1^ of soil) was higher than that of 3 other abandoned cropland soils (average numbers of copies·g^−1^ of soil, 8.2×10^7^ in Y1, 5.2×10^7^ in Y5, and 4.4×10^7^ in Y25) (Figure 3A and B). The 16S rRNA gene copy numbers did not change significantly over time in the abandoned cropland soils (P>0.05) (Figure 3B). Pearson's product-moment correlation indicated that NH_4_-N, organic C, total N, and plant biomass were positively correlated with the 16S rRNA gene copy number (Table 1).

### Effects of environmental variables on the microbial community structure

Because of multicollinearity among environmental variables (Table S3), potential effects of environmental variables on free-living nitrogen-fixing bacteria and the total bacteria community composition were assessed by PLSR (Table 2). Plant biomass, hydraulic conductivity, pH, Fe, and soil moisture, and NH4-N predominantly affected on both free-living nitrogen-fixing bacteria and the total bacteria community composition in this study area. Moreover, C and N also exerted effects on the total bacteria community composition (Table 2).

**Table 2:** Results of partial least squares regression (PLSR) analysis with the explanatory capacity of the first component and the variable influence on projection (VIP) of each predictor within each component to estimate significant predictors.

Gene	*nifH*	16S rRNA
Explained variance in fingerprinting pattern (%)	15.2	22.5
Explained variance of component predictors (%)	7.80	15.40

Environment variables*	Variable Influence on Projection (VIP)	
P-B	1.29	1.23
HC	1.17	1.07
pH	1.15	1.07
Fe	1.13	1.17
H_2_O	1.07	1.00
NH_4_-N	1.04	1.02
ELE	0.97	0.95
N	0.94	1.01
C	0.93	1.01
P	0.77	0.73
P-*H'*	0.66	0.75
NO_3_-N	0.61	0.70

*All environmental variables are shown and include pH, NO_3_-N, NH_4_-N, organic carbon (C), total nitrogen (N), available phosphorus (P), soluble iron (Fe), elevation (ELE), hydraulic conductivity (HC), soil moisture (H_2_O), plant biomass (P-B), and plant diversity (P-*H*′).

These correlations of microbial diversity with environmental parameters were also supported by RDA (Figure S2). The ordination plots for the *nifH* and 16S rRNA genes are given in Figure S2A and S2B, respectively. In these figures, the first 2 canonical axes explained 43.6% and 59.1% of the variance of the species data (functional genes) and 51.0% and 63.4% of the variance of the species–environment relationship, respectively. The projection of environmental variables with respect to the free-living nitrogen-fixing community composition revealed that the first canonical axis is positively correlated with soil moisture, Fe, NH4-N, and plant biomass, and it is negatively correlated with pH and hydraulic conductivity (Figure S2A). With respect to the total bacteria community composition, the first canonical axis is positively correlated with Fe, NH4-N, plant biomass, C, and N, and it is negatively correlated with pH and hydraulic conductivity (Figure S2B).

## Discussion

This study allowed a detailed analysis of the effects of key components of abandoned cropland with the same land use history (permanent grassland was turned into arable land by cultivation about 40 years ago and had rotated crops of *Zea mayscorn*, *Helianthus annuus* and *E. cylindricus*) and light-grazing steppe grassland systems on soil bacterial and free-living nitrogen-fixing bacterial population structure and gene copy number. Cultivation imparted a strong effect on both total and free-living nitrogen-fixing bacterial population structures (measured by DGGE profiles) and abundances (measured by gene copy numbers).

The copy numbers of *nifH* per gram of LGSG soil was higher than that in Y1, Y5, and Y25 soil. In this study, markedly lower levels of nutrient (e.g., organic C, P, and Fe) in the soil of abandoned cropland (Table 1) may suppress the growth of free-living nitrogen-fixing bacteria. Organic C, P, and Fe and *nifH* copy number have a significant positive Pearson's product-moment correlation (Table 1). The source of carbohydrate is important to allow N_2_ fixation activity, which requires large amounts of energy and reducing equivalents [49]. Phosphorus availability can increase nitrogen fixation because nitrogen fixation process requires large amounts of adenosine triphosphate (ATP) [50]. The [4Fe-4S] cluster of the *nifH* gene requires the Fe [22], [51]; therefore, a low copy number of *nifH* might be related to the content of organic C, P, and Fe. In addition, Coelho et al. [52], [53] found that 30% more free-living diazotrophs could be isolated from soil in the presence of low levels of nitrogen fertilizer than from soil in the presence of high levels of nitrogen fertilizer. In contrast, low copy number of *nifH* was obtained from the abandoned cropland of low nitrogen levels (NO_3_-N and NH_4_-N) (Table 1). The result may be caused by nutrients content of abandoned cropland soils.

Shannon-Wiener diversity index of *nifH* of LGSG was lower than that of Y1 and Y5 (Figures 2A and 3A). Previous studies indicated that pH is an important factor for the structure of the bacterial community [54], [55]. Belnap [56] thought that most nitrogen-fixing microorganisms have an optimum soil pH of 7 or above. In this study, the samples from abandoned croplands with pH>7 had a higher diversity of free-living nitrogen-fixing microorganisms than the samples from the LGSG with pH<7.

The *nifH* gene profile showed there was a higher variability between replicate samples than that of the 16S rRNA gene profile. Therefore, this result suggested that the free-living nitrogen-fixing bacteria community was susceptible in this study area. An explanation for this may be the local changes at the microsite/aggregate scale because of spatial and temporal variation in exudation along plant roots [57]–[59]. Similar approaches have demonstrated that there are differences in ammonia oxidizer populations in sediment and soil [60].

The RDA revealed that the distribution of the factors that influence both the free-living nitrogen-fixing bacteria and the broader bacterial community distribution are very similar. This suggests that soil factors that affect the free-living nitrogen fixers are likely to affect the community as a whole in steppe grassland soil. Larkin et al. [61] reported that plant effects are the most important drivers of soil microbial community characteristics within a given site and soil type. In our study, PLSR and RDA showed that plant biomass strongly influenced (Table 2) and was positively correlated with the first axis (Figure S2) in both the free-living nitrogen-fixing bacteria and the broader bacterial community distribution. This is caused by interactions between plants and microorganisms. Above-ground net primary productivity was expected to increase soil carbon input by enhancing the turnover of plant biomass and enhancing root exudation and may therefore influence carbon-limited microbial communities in the soil [62], [63]. Concurrently, microorganisms also affect plants by producing canonical plant growth-regulating substances such as auxins or cytokinins [13]. However, the Shannon-Wiener diversity index was not significantly influenced in either the free-living nitrogen-fixing bacteria or the broader bacterial community distribution in this study area. Pearson's product-moment correlations showed that plant diversity was not significantly correlated with *nifH* gene diversity, or 16S rRNA gene diversity and copy number, but did negatively correlate with the copy number of the *nifH* gene (Table 1). Carney and Matson [64] found that plant diversity had a significant effect on the microbial community composition through alterations in microbial abundance rather than community composition. However, several studies report that plant diversity has little direct effect on bacterial community composition [65], [66]. Therefore, this effect may depend on the type of plant community examined.

A number of additional factors such as hydraulic conductivity, pH, Fe, soil moisture, and NH4-N significantly influenced both the free-living nitrogen-fixing bacteria and the broader bacterial community distribution (Table 2). In our study, the soil from different sites was the same type, but the water holding capacity function of each research soil was different. The soil moisture content is in the order of LGSG>Y25>Y5>Y1 (Table 1). This suggests that a balance of macropores and micropores has not been fully recovered in the abandoned crop soils. A balance of macropores and micropores in soil influences the permeability and water holding capacity of soil [67]. In addition, the biological decomposition of organic materials produces natural glues, which bind and strengthen soil aggregates [67], and helps soils hold water and nutrients, which may change the balance of macropores and micropores. Organic matter also is a long-term, slow-release storehouse of nitrogen, phosphorus, and sulfur [67]. Accordingly, organic matter significantly influenced the total bacterial community distribution (Table 2), which may have resulted because of the significant difference in soil organic matter content in the abandoned cropland and LGSG (*P*<0.05) (Table 1). Additionally, the soil texture and hydraulic conductivity is closely correlated, and soil texture influences the balance between macropores and micropores [67]. The hydraulic conductivities of sites Y1 and Y5 were significantly different from those of sites Y25 and LGSG (Table 1), and the RDA also indicated that soil moisture was negatively correlated with hydraulic conductivity (Figure S2). Therefore, the hydraulic conductivity of soil also showed that the water holding capacity function of soils from the research sites is different.

The significance of NH4-N indicates that the fixed nitrogen of the free-living nitrogen-fixing bacteria will be the main nitrogen source in the soil of steppe grasslands, where there are few plants such as legumes. Nitrogen cycling in natural ecosystems and traditional agricultural production relies on biological nitrogen fixation primarily by diazotrophic bacteria [16].

In our study, higher copy number/diversity of the *nifH* gene/16S rRNA gene was observed in the LGSG soil, where the Fe content was significantly higher, than in the abandoned cropland soil. According to Pearson's product-moment correlation and PLSR, the Fe content affects the nitrogen-fixing community. This may result because Fe is a required material for the [4Fe-4S] cluster of the *nifH* gene [22], [51], and it is possible that the Fe content affects the 16S rRNA gene diversity in other microorganisms with the iron-containing enzyme [68]-[70] as it does the nitrogen-fixing bacteria.

Gaby and Buckley [71] comprehensively evaluated primers of *nifH* gene and found that *nifH* gene different primer sets amplified different groups in the *nifH* phylogeny. In this study, we used primer sets for DGGE and qPCR analyses of the *nifH* gene that targeted different sequence positions, thus the results obtained were not comparable. However, environmental factors differed greatly between the abandoned cropland and LGSG soils, thus having differential effects on the soil microbial communities. Furthermore, the trends in the changing copy number and diversity of the *nifH* gene were similar to that of the 16S rRNA gene. Therefore, the results we obtained regarding the copy number and diversity of *nifH* gene are able to explain the dynamic change in the trend of free-living nitrogen-fixing bacteria communities in abandoned cropland during different periods. Further research on molecular ecology could provide more detail analysis of microbial diversity, such as by sequencing of DGGE band and high-throughput sequencing technology etc., which would contribute to more accurate insights into the black box of the dynamic change of microbial communities.

We discovered that microbial communities of the abandoned cropland in this study area are strongly influenced by plant biomass, soil moisture, Fe, and NH4-N. Robust information about the mechanisms that regulate the diversity, structure, and composition of natural communities is urgently needed to help conserve ecosystem function and mitigate biodiversity loss from current and future environmental changes. Conversely, the results of this study suggest that advances in desertification may be prevented by adjusting environmental factors of the abandoned cropland, such as the soil moisture content, Fe, and NH4-N, which will enhance the function of a microbial community and possibly increasing plant biomass production. In addition, *nifH* gene copy number had significant positive Pearson's product-moment correlation with soil moisture, organic C, P, and Fe etc., therefore, by adjusting these environmental factors may also increase the abundance of free-living nitrogen-fixing bacteria in abandoned cropland.

## Supporting Information

Figure S1 **Denaturing gradient gel electrophoresis (DGGE) profiles of the** ***nifH*** **(A) and 16S rRNA genes (B).** For all images, the numbers refer to the plot numbers in the sample areas.
(DOCX)

Figure S2 **Redundancy analysis (RDA) of** ***nifH*** **(A) and 16S rRNA (B) genes data.** Ordination plots of *nifH* (A) and 16S rRNA (B) genes associated with abandoned croplands for different times: Y1 (•), Y5 (▴), and Y25 (▪), and *light-grazing* steppe grassland (LGSG, ○). The plots were generated by redundancy analysis (RDA) of the denaturing gradient gel electrophoresis (DGGE) profiles. All environmental variables are shown, including pH, NO_3_-N, NH_4_-N, organic carbon (C), total nitrogen (N), available phosphorus (P), soluble iron (Fe), elevation (ELE), hydraulic conductivity (HC), soil moisture (H_2_O), plant biomass (P-B), and plant species richness (P-*H*′). Values on the axes indicate the percentages of total variation explained by each axis.
(DOCX)

Table S1 Total plant cover, plant Shannon's diversity index, plant biomass, plant types, and their coverage in each research plot.
(DOCX)

Table S2 Soil texture (clay, silt, and sand) content and saturated hydraulic conductivity of soil samples at each site.
(DOCX)

Table S3 Collinearity among environmental parameters as determined by Spearman's rank correlation coefficient rho. Significantly correlated parameters show Spearman's rank correlation coefficient rho>0.6 and <−0.6 in bold. All environmental variables are shown, such as pH, NO_3_-N, NH_4_-N, organic carbon (C), total nitrogen (N), available phosphorus (P), soluble iron (Fe), elevation (ELE), hydraulic conductivity (HC), soil moisture (H_2_O), plant biomass (P-B), and plant diversity (P-*H*′).
(DOCX)
