# Supplementary material for: Effect of Abandonment on Diversity and Abundance of Free-Living Nitrogen-Fixing Bacteria and Total Bacteria in the Cropland Soils of Hulun Buir, Inner Mongolia
Source: PLoS One. 2014 Sep 30;9(9):e106714. doi: 10.1371/journal.pone.0106714 (PMC4182089; doi:10.1371/journal.pone.0106714)
Supplement: Figure S1 — Denaturing gradient gel electrophoresis (DGGE) profiles of the nifH (A) and 16S rRNA genes (B). For all images, the numbers refer to the plot numbers in the sample areas. (DOCX) [file pone.0106714.s001.docx]

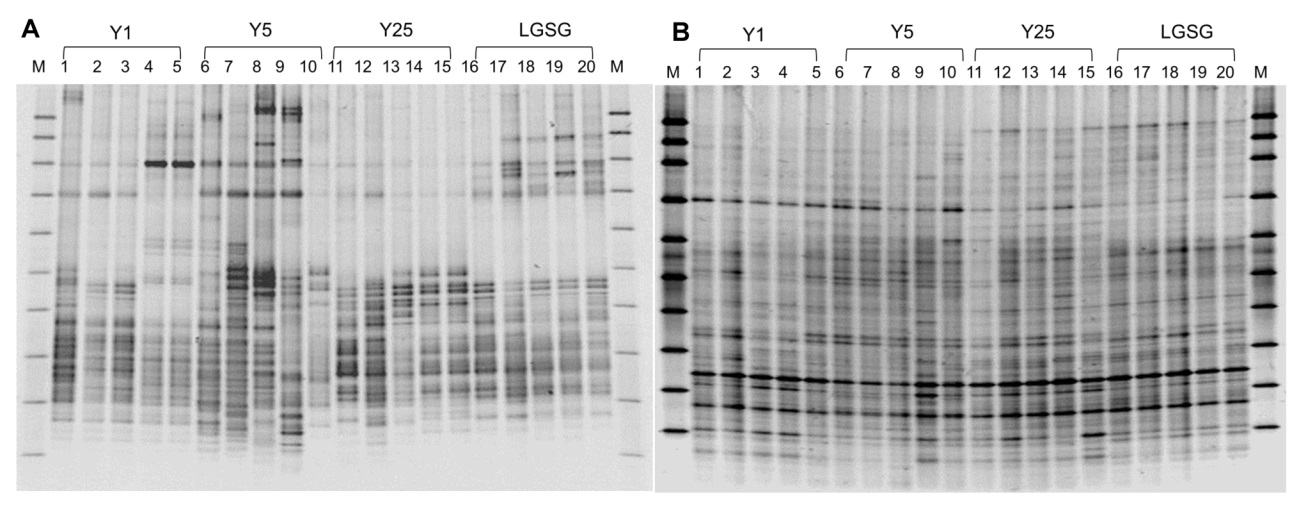


**Figure S1. Denaturing gradient gel electrophoresis (DGGE) profiles of the *nifH* (A) and 16S rRNA genes (B). For all images, the numbers refer to the plot numbers in the sample areas.**
