# Supplementary material for: Effect of Abandonment on Diversity and Abundance of Free-Living Nitrogen-Fixing Bacteria and Total Bacteria in the Cropland Soils of Hulun Buir, Inner Mongolia
Source: PLoS One. 2014 Sep 30;9(9):e106714. doi: 10.1371/journal.pone.0106714 (PMC4182089; doi:10.1371/journal.pone.0106714)
Supplement: Figure S2 — Redundancy analysis (RDA) of nifH (A) and 16S rRNA (B) genes data. Ordination plots of nifH (A) and 16S rRNA (B) genes associated with abandoned croplands for different times: Y1 (•), Y5 (▴), and Y25 (▪), and light-grazing steppe grassland (LGSG, ○). The plots were generated by redundancy analysis (RDA) of the denaturing gradient gel electrophoresis (DGGE) profiles. All environmental variables are shown, including pH, NO3-N, NH4-N, organic carbon (C), total nitrogen (N), available phosphorus (P), soluble iron (Fe), elevation (ELE), hydraulic conductivity (HC), soil moisture (H2O), plant biomass (P-B), and plant species richness (P-H′). Values on the axes indicate the percentages of total variation explained by each axis. (DOCX) [file pone.0106714.s002.docx]

**
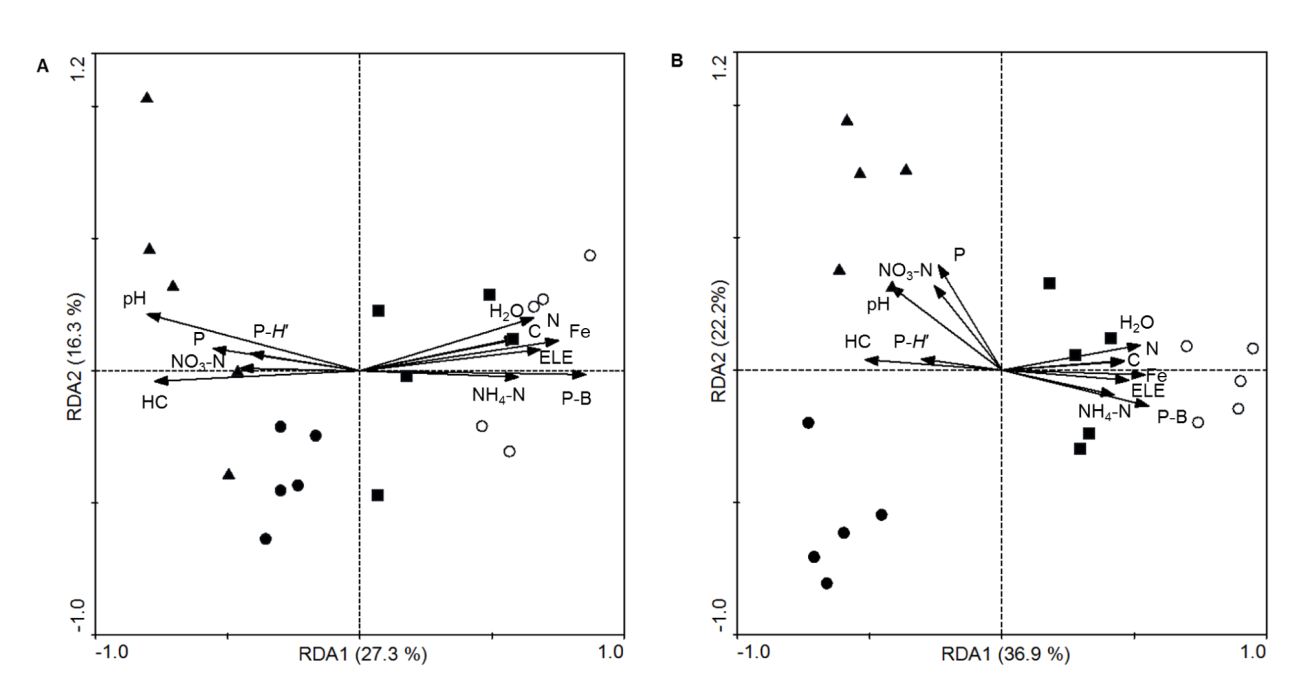
**

**Figure S2. Redundancy analysis (RDA) of *nifH* (A) and 16S rRNA (B) genes data. Ordination plots of *nifH* (A) and 16S rRNA (B) genes associated with abandoned croplands for different times: Y1 (●), Y5 (▲), and Y25 (■), and *light-grazing* steppe grassland (LGSG, ○). The plots were generated by redundancy analysis (RDA) of the denaturing gradient gel electrophoresis (DGGE) profiles. All environmental variables are shown, including** **pH, NO_3_-N, NH_4_-N, organic carbon (C), total nitrogen (N), available phosphorus (P), soluble iron (Fe), elevation (ELE), hydraulic conductivity (HC), soil moisture (H_2_O), plant biomass (P-B), and plant species richness (P-*H*′). Values on the axes indicate the percentages of total variation explained by each axis.**
