# Supplementary material for: Effect of Abandonment on Diversity and Abundance of Free-Living Nitrogen-Fixing Bacteria and Total Bacteria in the Cropland Soils of Hulun Buir, Inner Mongolia
Source: PLoS One. 2014 Sep 30;9(9):e106714. doi: 10.1371/journal.pone.0106714 (PMC4182089; doi:10.1371/journal.pone.0106714)
Supplement: Table S1 — Total plant cover, plant Shannon's diversity index, plant biomass, plant types, and their coverage in each research plot. (DOCX) [file pone.0106714.s003.docx]

**Table S1. Total plant cover, plant Shannon’s diversity index, plant biomass, plant types, and their coverage in each research plot.**

| **Abandoned cropland** | **Y1** | | | | | **Y5** | | | | | **Y25** | | | | | **LGSG** | | | | |
| --- | --- | --- | --- | --- | --- | --- | --- | --- | --- | --- | --- | --- | --- | --- | --- | --- | --- | --- | --- | --- |
| Plot No. | 1 | 2 | 3 | 4 | 5 | 6 | 7 | 8 | 9 | 10 | 11 | 12 | 13 | 14 | 15 | 16 | 17 | 18 | 19 | 20 |
| Total cover (%) | 30 | 45 | 40 | 35 | 35 | 15 | 20 | 22 | 12 | 25 | 40 | 38 | 35 | 45 | 50 | 50 | 50 | 60 | 65 | 55 |
| Shannon's diversity index (P-*H*') | 2.04 | 1.57 | 1.57 | 1.90 | 1.07 | 1.33 | 2.54 | 1.61 | 1.59 | 1.48 | 1.72 | 1.56 | 1.75 | 1.88 | 2.20 | 0.93 | 0.93 | 0.98 | 0.93 | 0.93 |
| Plant biomass (g/m^2^) | 40.50 | 59.72 | 50.14 | 57.77 | 34.38 | 11.88 | 25.14 | 18.04 | 15.49 | 15.25 | 94.36 | 97.21 | 66.21 | 65.92 | 74.58 | 146.73 | 132.56 | 148.32 | 141.78 | 140.56 |
| *Convolvulus ammannii* |  |  |  |  |  |  |  |  |  |  |  |  |  |  |  | + | + | + |  | + |
| *Agropyron cristatum* |  |  |  |  |  |  |  |  |  |  |  |  |  |  |  | + | + | + | + | + |
| *Allium mongolicum* |  |  |  |  |  |  |  |  |  |  |  |  |  |  |  | + | + | + | + | + |
| *Stipa krylovii* |  |  |  |  |  |  |  | + |  |  | 1 | 1 | 1 | 1 | 2 | 2 | 2 | 2 | 2 | 2 |
| *Leymus chinensis* |  |  |  |  |  |  | 1 |  |  |  | + | 1 |  | 2 | 1 | 1 | 1 | 1 | 1 | 1 |
| *Potentilla bifurca* |  |  |  |  |  | + | + | + |  |  |  |  |  |  |  | + | + | + | + |  |
| *Carex korshinskyi* |  |  |  |  |  | 1 | 1 | 1 | 1 | 1 |  |  |  |  |  | + | + | + | + | + |
| *Artemisia frigida* |  |  |  |  |  |  |  |  |  |  | 1 | 1 | 1 | 1 | 1 |  |  |  |  |  |
| *Allium ramosum* |  |  |  |  |  |  |  |  |  |  | 1 | 1 | 1 | 1 | 1 |  |  |  |  |  |
| *Cleistogenes squarrosa* |  |  |  |  |  |  |  |  |  |  | 1 |  | + | 1 | 1 |  |  |  |  |  |
| *Artemisia eriopoda* |  |  |  |  |  | 1 | 1 | 1 | 1 | 2 | 2 | 2 | 2 | 3 | 3 |  |  |  |  |  |
| *Salsola collina* | 1 | 2 | + | 1 | + | + | 1 | + | 1 | 1 |  |  | + |  | + |  |  |  |  |  |
| *Chenopodium acuminatum* | 1 | 1 | 1 | + | + |  |  | + | + | + |  |  |  |  | + |  |  |  |  |  |
| *Setaria viridis* | 1 | 1 | 1 | 1 | 1 |  | 1 | 1 | 1 | 1 |  |  |  |  |  |  |  |  |  |  |
| *Eragrostis pilosa* | 2 | 2 | 2 | 2 | 2 |  | + |  |  |  |  |  |  |  |  |  |  |  |  |  |
| *Polygonum bungeanum* | + |  | + | 1 |  |  |  |  |  |  |  |  |  |  |  |  |  |  |  |  |
| *Erodium stephanianum* | 1 | 1 | 1 | 1 | 1 |  |  |  |  |  |  |  |  |  |  |  |  |  |  |  |
| *Calystegia hederacea* |  | 1 | 1 | 1 |  |  |  |  |  |  |  |  |  |  |  |  |  |  |  |  |
| *Artemisia annua* |  |  | + | 1 | + |  |  |  |  |  |  |  |  |  |  |  |  |  |  |  |

**Cover classes: 3, coverage (%) between 25 and 50; 2, coverage (%) between 10 and 25; 1, coverage (%) between 1 and 10; +, coverage (%) below 1. Vacant places in the table indicate that the plant was not found in the corresponding plot**
