# Supplementary material for: Effect of Abandonment on Diversity and Abundance of Free-Living Nitrogen-Fixing Bacteria and Total Bacteria in the Cropland Soils of Hulun Buir, Inner Mongolia
Source: PLoS One. 2014 Sep 30;9(9):e106714. doi: 10.1371/journal.pone.0106714 (PMC4182089; doi:10.1371/journal.pone.0106714)
Supplement: Table S2 — Soil texture (clay, silt, and sand) content and saturated hydraulic conductivity of soil samples at each site. (DOCX) [file pone.0106714.s004.docx]

**Table S2.** **Soil texture (clay, silt, and sand) content and saturated hydraulic conductivity of soil samples at each site.**

|  |  | **Soil texture (%)** | | |  |
| --- | --- | --- | --- | --- | --- |
| **Sites** | **Classification** | **clay** | **silt** | **sand** | **Hydraulic Conductivity (×10^-3^ cm^-1^)** |
| Y1 | Sandy loam | 13.37±0.54 | 9.82±1.44 | 76.81±1.95 | 2.93±0.07 |
| Y5 | Sandy loam | 11.96±0.32 | 11.23±0.46 | 76.81±0.72 | 2.95±0.03 |
| Y25 | Sandy loam | 14.94±1.2 | 11.81±1.33 | 73.25±0.45 | 2.79±0.02 |
| LGSG | Sandy loam | 13.88±0.36 | 14.49±0.26 | 71.64±0.45 | 2.75±0.02 |

**The soil texture was classified according to the International Society of Soil Science (ISSS) classification system.**
