# Supplementary material for: Effect of Abandonment on Diversity and Abundance of Free-Living Nitrogen-Fixing Bacteria and Total Bacteria in the Cropland Soils of Hulun Buir, Inner Mongolia
Source: PLoS One. 2014 Sep 30;9(9):e106714. doi: 10.1371/journal.pone.0106714 (PMC4182089; doi:10.1371/journal.pone.0106714)
Supplement: Table S3 — Collinearity among environmental parameters as determined by Spearman's rank correlation coefficient rho. Significantly correlated parameters show Spearman's rank correlation coefficient rho>0.6 and <−0.6 in bold. All environmental variables are shown, such as pH, NO3-N, NH4-N, organic carbon (C), total nitrogen (N), available phosphorus (P), soluble iron (Fe), elevation (ELE), hydraulic conductivity (HC), soil moisture (H2O), plant biomass (P-B), and plant diversity (P-H′). (DOCX) [file pone.0106714.s005.docx]

**Table S3. Collinearity among environmental parameters as determined by** **Spearman’s rank correlation coefficient rho. Significantly correlated parameters show Spearman’s rank correlation coefficient rho > 0.6 and < -0.6 in bold. All environmental variables are shown, such as pH, NO_3_-N, NH_4_-N, organic carbon (C), total nitrogen (N), available phosphorus (P), soluble iron (Fe), elevation (ELE), hydraulic conductivity (HC), soil moisture (H_2_O), plant biomass (P-B), and plant diversity (P-*H*′).**

|  | ELE | HC | P-*H*′ | P-B | H2O | NO3-N | NH4-N | Fe | P | C | N | pH |
| --- | --- | --- | --- | --- | --- | --- | --- | --- | --- | --- | --- | --- |
| ELE | **1.000** |  |  |  |  |  |  |  |  |  |  |  |
| HC | -0.587 | **1.000** |  |  |  |  |  |  |  |  |  |  |
| P-*H*′ | -0.574 | 0.497 | **1.000** |  |  |  |  |  |  |  |  |  |
| P-B | **0.710** | **-0.808** | -0.423 | **1.000** |  |  |  |  |  |  |  |  |
| H2O | 0.250 | **-0.780** | -0.477 | **0.716** | **1.000** |  |  |  |  |  |  |  |
| NO3-N | -0.205 | 0.332 | -0.241 | -0.523 | -0.226 | **1.000** |  |  |  |  |  |  |
| NH4-N | **0.821** | -0.574 | -0.552 | **0.724** | 0.385 | -0.286 | **1.000** |  |  |  |  |  |
| Fe | 0.443 | **-0.672** | -0.383 | **0.809** | **0.779** | -0.334 | 0.495 | **1.000** |  |  |  |  |
| P | -0.534 | 0.215 | -0.030 | -0.544 | -0.095 | 0.535 | -0.379 | -0.352 | **1.000** |  |  |  |
| C | 0.326 | -0.531 | **-0.614** | 0.374 | 0.587 | 0.370 | 0.412 | 0.571 | 0.244 | **1.000** |  |  |
| N | 0.286 | -0.468 | **-0.630** | 0.320 | 0.572 | 0.390 | 0.425 | 0.547 | 0.249 | **0.976** | **1.000** |  |
| pH | **-0.775** | **0.777** | 0.491 | **-0.933** | **-0.606** | 0.495 | **-0.753** | **-0.778** | **0.639** | -0.313 | -0.298 | **1.000** |
